# Equation-Based Modeling of Shape Memory Alloys for Reinforcement of Masonry Structures Against Out-of-Plane Excitation

**DOI:** 10.3390/ma18133124

**Published:** 2025-07-01

**Authors:** Kacper Wasilewski, Artur Zbiciak, Wojciech Terlikowski

**Affiliations:** Faculty of Civil Engineering, Warsaw University of Technology, al. Armii Ludowej 16, 00-637 Warsaw, Poland; artur.zbiciak@pw.edu.pl (A.Z.); wojciech.terlikowski@pw.edu.pl (W.T.)

**Keywords:** superelasticity, rheological models, seismic retrofitting of masonry structures

## Abstract

The incorporation of advanced smart materials, such as shape memory alloys (SMAs), in civil engineering presents significant challenges, particularly in modeling their complex behavior. Traditional numerical SMA models often require material parameters that are difficult to estimate and validate. The objective of this paper is to introduce an equation-based approach to modeling the superelastic behavior of SMAs based on rheological models. The proposed phenomenological model accurately captures SMA superelasticity under isothermal conditions, with each material parameter directly correlated to data from standard mechanical experiments. Four modifications to the baseline rheological model are proposed, highlighting their impact on superelastic characteristics. The resulting constitutive relationships are expressed as non-linear ordinary differential equations, making them compatible with commercial finite element method (FEM) software through user-defined subroutines. The practical application of this modeling approach is demonstrated through the strengthening of a historical masonry wall subjected to seismic activity. Comparative analysis shows that ties incorporating SMA segments outperform traditional steel ties by reducing the potential damage and enhancing the structural performance. Additionally, the energy dissipation during the SMA phase transformation improves the damping of vibrations, further contributing to the stability of the structure. This study underscores the potential of SMA-based solutions in seismic retrofitting and highlights the advantages of equation-based modeling for practical engineering applications.

## 1. Introduction

The mathematical modeling and numerical implementation of superelasticity and shape memory effects in shape memory alloys (SMAs) have been extensively studied over the past decades. A key distinction between various mathematical models lies in their reference scale. The most precise models describe microstructural phenomena such as nucleation, phase transition, and twinned martensite growth at the single-crystal level. These include models based on Ginzburg–Landau superconductivity theory (e.g., Falk’s model [1] or Jin et al. [2]) and molecular dynamics (e.g., Lai and Liu [3] or Kastner [4]). Due to their computational intensity, such models are suitable for microstructural analysis but not for structural simulations. A second category comprises micro/macro models. They describe the behavior of the material at the micro or meso scale, which is then upscaled to derive macroscopic constitutive equations. The scaling process can be performed, for example, using the Mori–Tanaka approximation (Siredey et al. [5]) or the self-consistent scheme (Patoor et al. [6]). These models allow for highly accurate material simulations; however, a significant limitation is the need to determine numerous material parameters, which can be challenging [7]. The final category consists of macroscopic models that describe the behavior of polycrystalline SMA using phenomenological approaches, simplified micro/macro thermodynamics, or direct fitting to experimental data. A comprehensive review of SMA material models and their historical development can be found in the works of Lagoudas [8], Cisse et al. [7], and Marfia and Vigliotti [9]. The study by Tabrizikahou et al. [10] discusses SMA applications in structural vibration simulations and the numerical models commonly used for seismic analysis.

The use of SMAs’ specific characteristics, such as superelasticity, in the strengthening of existing and new buildings has been a topic of theoretical and practical research for decades. The numerical simulation, which is presented in this paper to demonstrate the possible application of a phenomenological modeling approach, is based on existing applications of SMA in the Basilica of St. Francis in Assisi [11], the Cathedral of St. Felician in Foligno [12], and the Church of St. Seraphin in Montegranaro [13]. More recent studies on SMA strengthening of historical buildings also address one of its largest limitations, which is the cost of materials [14,15]. Authors underline the cost-effectiveness of SMA implementation in case of applications of elements that require no customization or machining. Most of the studies and applications present the most widespread SMA material, which is an alloy of nickel and titanium, but other alloys, for instance based on copper (Cu-Al-Be), have also been studied and applied as part of ties in historical structures [16].

The advantages of SMA applications for seismic retrofitting have been proven by numerical and laboratory investigations. The study [17] demonstrates the ability of superelastic SMA ties and reinforcing strategies to significantly improve seismic performance in vulnerable masonry heritage structures. Using numerical models, the authors show that SMA provides strong self-centering behavior and energy dissipation, reducing residual displacements and facilitating structural recovery after seismic events. The investigations of SMA applications are not limited to masonry but also more recent reinforced concrete structures. The paper [18] investigates full-scale RC building models retrofitted with SMA buckling-restrained braces. Time-history analyses show that SMA-based braces are highly effective at limiting both peak inter-story drifts and residual deformations due to their excellent energy dissipation and recentering capabilities. The application of SMA for vibration damping is not limited to ties or tie-like; other elements are also investigated. The study [19] summarizes advancements in SMA core damping technologies, including wires, bars, cables, and rings. It highlights both full-scale and proof-of-concept experimental validations. The work explores the benefits of these elements, such as energy dissipation, recentering, and mechanical reliability, and outlines areas needing further development.

The aim of this paper is to introduce superelasticity equation-based modeling of SMA reinforcement of masonry against out-of-plane excitation. The presented modeling strategy is based on rheological models that represent the superelasticity phenomena of SMA in isothermal conditions, as already introduced in [20,21]. To verify this phenomenological modeling approach, an example of a theoretical case study is presented. SMA ties are used as a strengthening technique for masonry tympanum subjected to earthquake excitation. The presented results are in agreement with the phenomenological concept of the modeling approach and reveal the advantages of application of this strengthening technique, such as the controllable punctual force transferred to the masonry structure.

## 2. SMA Modeling with Rheological Structures

**Figure 1:**
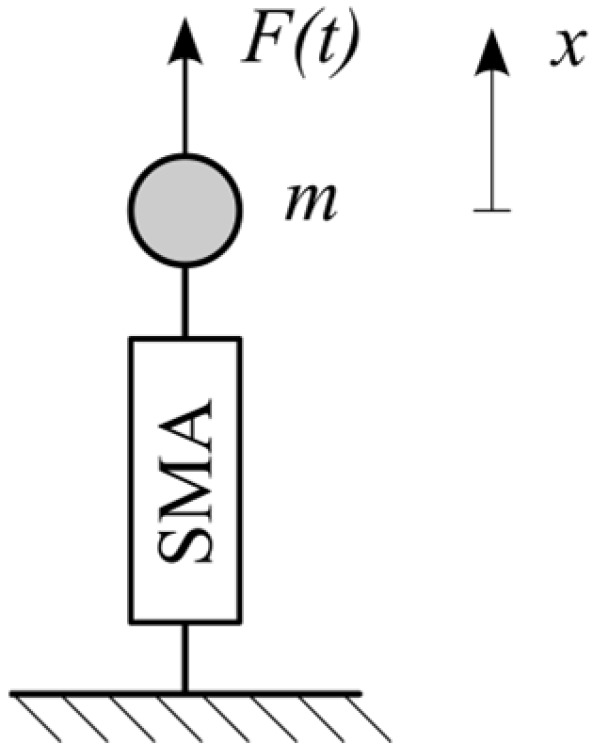
Dynamic SMA model—single-degree-of-freedom oscillator scheme.

To formulate a basic dynamic model incorporating an SMA structure, a single-degree-of-freedom oscillator was considered (Figure 1). The phenomenological SMA model is represented graphically as a so-called black box. The paper describes the rheological structures composed of elements that model SMA behavior under isothermal conditions, expanding on the black box concept.

Figure 1 illustrates a basic single-degree-of-freedom dynamic oscillator incorporating an SMA element represented as a rheological black box. This schematic provides a clear physical interpretation of the SMA model, linking the displacement, mass, and reaction forces within the model. The equation of motion for the considered oscillator is a second-order differential equation:

(1) $$\ddot{x}\left(t\right)=\frac{1}{m}[F\left(t\right)-S\left(t\right)],$$

where $F\left(t\right)$ is the external excitation, $S\left(t\right)$ is the reaction force of the rheological structure, and x is the displacement of a material point with mass m. Equation (1) has a universal form and will be used to describe all rheological structures presented below.

### 2.1. Basic Model

**Figure 2:**
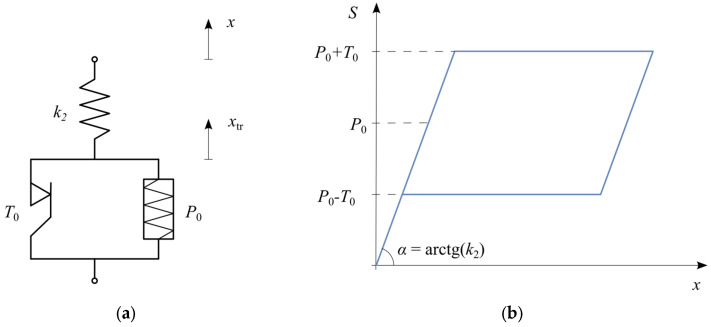
Basic SMA model: (**a**) rheological structure; (**b**) hysteresis loop.

The basic rheological structure of an SMA (Figure 2a) consists of a spring $k_{2}$ connected in series with a slider $T_{0}$, and a pre-stressed spring $P_{0}$ in parallel arrangement. A cyclic tensile test of this structure results in a hysteresis loop (Figure 2b) with a flag-shaped curve characteristic of superelasticity.

At the initial loading stage, the displacement of the mass m increases linearly, depending on the stiffness of the spring $k_{2}$. When the force reaches $P_{0}+T_{0}$, a plateau characteristic of the martensitic transformation appears. During unloading, the hysteresis loop follows a parallel path, again featuring a plateau, this time corresponding to the reverse phase transformation at force $P_{0}-T_{0}$. Further unloading restores the displacement to zero, meaning full shape recovery.

The structure of the basic SMA model is shown in Figure 2a. It consists of a spring in series with a slider, accompanied by a pre-stressed parallel spring. The resulting hysteresis loop, characteristic of superelasticity, is depicted in Figure 2b, clearly showing the distinctive plateau regions corresponding to phase transformation. The internal force S in this SMA model depends on the displacement x (displacement of the material point with mass m, Figure 1) and the displacement due to phase transformation $x_{tr}$, given by

(2) $$S(t)=k_{2}\left(x(t)-x_{tr}(t)\right).$$

The ratio of the phase transformation displacement ${\dot{x}}_{tr}$ is expressed as a characteristic function $f_{SMA}$ in the relations:

(3) $$\begin{array}{l} {\dot{x}}_{tr}=f_{SMA}\left(\dot{x},x,x_{tr}\right)\\ f_{SMA}=\left\{\begin{array}{c} \dot{x}\;\mathrm{if}\;\left|S\right|=P_{0}+T_{0}\;\mathrm{and}\;S\dot{x}>0\\ \dot{x}\;\mathrm{if}\;\left|S\right|=P_{0}-T_{0}\;\mathrm{and}\;S\dot{x}<0\;\mathrm{and}\;Sx_{tr}>0\\ 0\;\mathrm{otherwise} \end{array}\right. \end{array}$$

The above conditional logic Equation (3) determines whether the SMA element is actively undergoing phase transformation. The first condition corresponds to initiating the martensitic transformation (loading plateau), the second condition corresponds to the reverse transformation (unloading plateau), and the third condition represents no transformation occurring.

Solving the non-linear system of differential equations allows determining the oscillator displacement $x\left(t\right)$ in response to a given excitation F(t).

### 2.2. Model with Hardening During Phase Transformation

**Figure 3:**
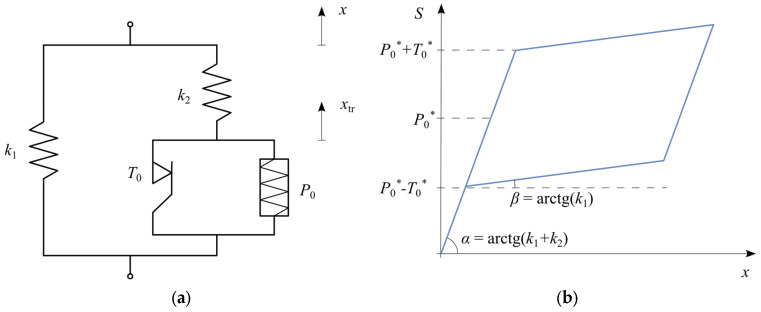
SMA model incorporating hardening during the martensitic transformation: (**a**) rheological structure; (**b**) hysteresis loop.

By modifying the basic SMA model with an additional parallel spring, an effect corresponding to hardening during the martensitic transformation was achieved. This modification alters the hysteresis loop—unlike the basic model, the phase transformation plateaus are no longer horizontal (Figure 3). Moreover, due to the kinematic hardening effect associated with the additional spring $k_{1}$, the critical forces initiating the phase transformations ($P_{0}$ and $T_{0}$) must be modified according to Equation (4).

(4) $$\begin{array}{cc} P_{0}^{*}=\frac{k_{1}+k_{2}}{k_{2}}P_{0}, & T_{0}^{*}=\frac{k_{1}+k_{2}}{k_{2}}T_{0} \end{array}.$$

The figure clearly presents the modified rheological structure incorporating an additional spring to simulate kinematic hardening during martensitic transformation (Figure 3a). The corresponding hysteresis loop (Figure 3b) illustrates inclined plateaus, demonstrating how the stiffness increases gradually due to this hardening effect.

This structural modification retains the oscillator’s equation of motion (1) but requires modifying Equation (2) describing the internal force–displacement relationship to Equation (5).

(5) $$\begin{array}{l} S\left(t\right)=S_{1}\left(t\right)+S_{2}\left(t\right)\\ S_{1}\left(t\right)=k_{1}x\left(t\right)\\ S_{2}\left(t\right)=k_{2}\left(x\left(t\right)-x_{tr}\left(t\right)\right) \end{array}$$

Additionally, in the equation for the phase transformation displacement ratio (3), the force term S must be replaced by $S_{2}$, leading to Equation (6).

(6) $$\begin{array}{l} {\dot{x}}_{tr}=f_{SMA}\left(\dot{x},x,x_{tr}\right)\\ f_{SMA}=\left\{\begin{array}{c} \dot{x}\;\mathrm{if}\;\left|S_{2}\right|=P_{0}^{*}+T_{0}^{*}\;\mathrm{and}\;S_{2}\dot{x}>0\\ \dot{x}\;\mathrm{if}\;\left|S_{2}\right|=P_{0}^{*}-T_{0}^{*}\;\mathrm{and}\;S_{2}\dot{x}<0\;\mathrm{and}\;S_{2}x_{tr}>0\\ 0\;\mathrm{otherwise} \end{array}\right. \end{array}$$

The conditional logic Equation (6) governs the displacement due to phase transformation when the kinematic hardening effects are considered. The first condition corresponds to the start of the martensitic transformation (loading path), the second condition corresponds to the reverse transformation (unloading path), and the third condition indicates no ongoing phase transformation.

### 2.3. Model with Locking

**Figure 4:**
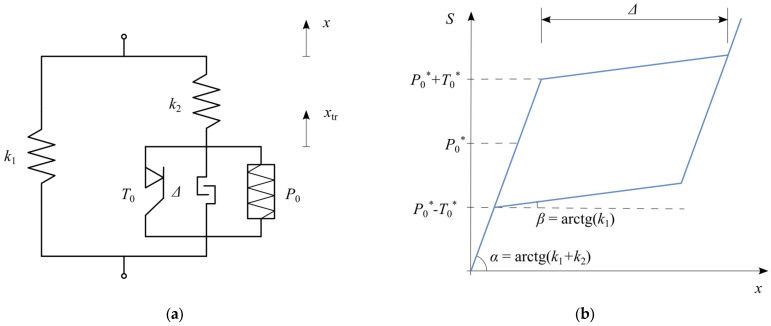
SMA model with locking: (**a**) rheological structure; (**b**) hysteresis loop.

Another modification introduces a locking effect to restore the material’s stiffness after the phase transformation is fully completed. This is achieved by incorporating a locking element that becomes fixed when the displacement reaches Δ (Figure 4).

The value of this displacement is a characteristic property of different shape memory alloys, usually defined as the strain level at which superelasticity occurs. Unlike the other phenomenological models [7], where phase transformation is considered complete when the martensite volume fraction reaches ξ=1, in this model, completion is defined by reaching a specific displacement threshold.

This figure illustrates the SMA model enhanced with a locking mechanism, where Figure 4a shows the modified structure incorporating a displacement-limiting component. The resulting hysteresis loop in Figure 4b distinctly depicts the material’s stiffness increase after the superelastic displacement threshold (locking displacement delta) is reached.

In this model, the displacement Δ applies only to the branch responsible for the superelastic behavior ($x_{tr}$). This necessitates modifying Equation (6) to Equation (7).

(7) $$\begin{array}{l} {\dot{x}}_{tr}=f_{SMA}\left(\dot{x},x,x_{tr}\right)\\ f_{SMA}=\left\{\begin{array}{c} \dot{x}\;\mathrm{if}\;\left|S_{2}\right|=P_{0}^{*}+T_{0}^{*}\;\mathrm{and}\;S_{2}\dot{x}>0\;\mathrm{and}\;\left|x_{tr}\right|<\Delta \\ \dot{x}\;\mathrm{if}\;\left|S_{2}\right|=P_{0}^{*}-T_{0}^{*}\;\mathrm{and}\;S_{2}\dot{x}<0\;\mathrm{and}\;S_{2}x_{tr}>0\\ 0\;\mathrm{otherwise} \end{array}\right. \end{array}$$

The condition set (7) clarifies how the locking displacement threshold is integrated into the model. The first condition defines the initiation and continuation of martensitic transformation until the displacement threshold is reached. The second condition captures the reverse transformation process. The final condition encompasses situations where no transformation occurs, or the locking limit has been reached.

### 2.4. Model with Different Crystal Phase Stiffnesses

**Figure 5:**
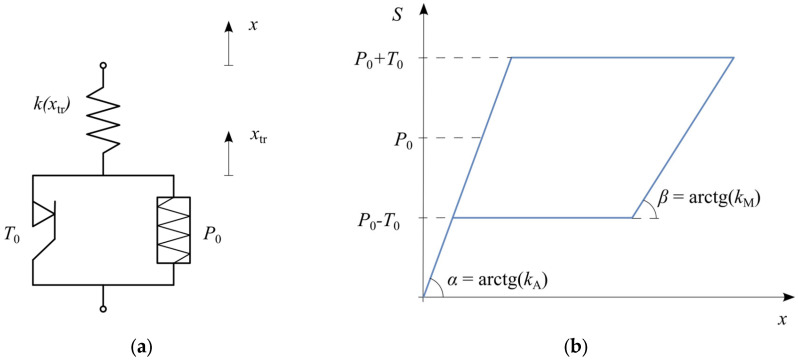
SMA model with different crystal phase stiffnesses: (**a**) rheological structure; (**b**) hysteresis loop.

This section introduces an original modification, proposed by the authors, to the basic SMA model, in which the spring stiffness is allowed to vary explicitly as a function of the phase transformation displacement $x_{tr}$. This approach captures the effect of different material stiffnesses in distinct phases (Figure 5b).

The schematic (Figure 5a) introduces a rheological structure explicitly accounting for different stiffness values of martensitic and austenitic phases. The hysteresis loop (Figure 5b) reveals how the stiffness variation impacts the response, with smooth transitions between phases clearly highlighted.

**Figure 6:**
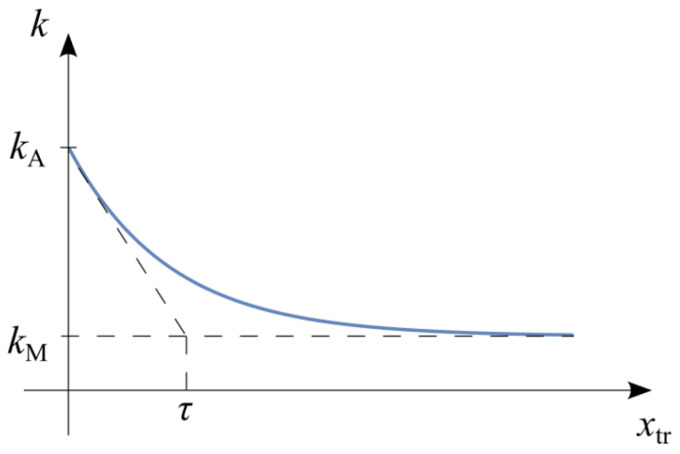
Assumed evolution of stiffness k as a function of phase transformation displacement $x_{tr}$.

A key assumption is defining the dynamics of the phase transformation. In classical models [7], the phase transformation is characterized by the martensite volume fraction ξ. The present model assumes an exponential phase transformation dynamic (Figure 6), with the spring stiffness function given by the following equation.

(8) $$k\left(x_{tr}\right)=k_{M}+\left(k_{A}-k_{M}\right)\exp\left(-\frac{\left|x_{tr}\right|}{\tau }\right),$$

where $k_{M}$ and $k_{A}$ are the stiffness values in the martensite and austenite phases, respectively, and τ is a parameter characterizing the transformation dynamics, analogous to an exponential time constant, representing the strain level $x_{tr}$ at which a fraction $\frac{1}{e}\cdot 100\%\approx 36.8\%$ of the material has transformed.

Figure 6 explicitly illustrates the assumed exponential variation in the stiffness as a function of the phase transformation displacement. It clarifies the physical interpretation of the parameter tau, which characterizes the transformation dynamics by marking the displacement at approximately 36.8% transformation completion.

Introducing a variable-stiffness spring into the rheological structure necessitates modifying Equations (2) and (3) to Equation (9).

(9) $$\begin{array}{l} S=k\left(x_{tr}\right)\left(x-x_{tr}\right)\\ {\dot{x}}_{tr}=k_{mod}\cdot f_{SMA}\left(\dot{x},x,x_{tr}\right) \end{array},$$

where $k_{mod}$ is a modifying coefficient determined by Equation (10).

(10) $$k_{mod}=\frac{k\left(x_{tr}\right)}{k\left(x_{tr}\right)-\frac{\partial k\left(x_{tr}\right)}{\partial x_{tr}}\left(x-x_{tr}\right)}.$$

After appropriate transformations, the modifying coefficient is given by the Equation (11).

(11) $$k_{mod}=\frac{k_{M}+\left(k_{A}-k_{M}\right)\cdot \exp\left(-\frac{\left|x_{tr}\right|}{\tau }\right)}{k_{M}+\left(k_{A}-k_{M}\right)\cdot \exp\left(-\frac{\left|x_{tr}\right|}{\tau }\right)+\frac{\left(k_{A}-k_{M}\right)\mathrm{sgn}\left(x_{tr}\right)}{\tau }\cdot \exp\left(-\frac{\left|x_{tr}\right|}{\tau }\right)\cdot \left(x-x_{tr}\right)}.$$

### 2.5. Model with Internal Hysteresis Loops

**Figure 7:**
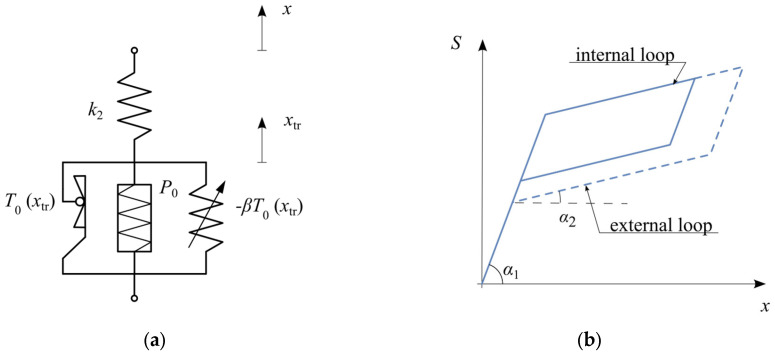
SMA model with internal hysteresis loops: (**a**) rheological structure; (**b**) hysteresis loop.

The phenomenon of internal hysteresis loops is characteristic of some shape memory alloys. It manifests as a change in the force level required to initiate the reverse phase transformation when the material has not undergone a complete transformation. For instance, if unloading occurs during the austenite-to-martensite transformation, the initial response is elastic, and it is followed by the characteristic plateau. However, the force level at which the reverse transformation begins is higher than in the case of complete transformation (Figure 7b).

To incorporate this effect, the basic SMA model requires modification. The classical slider, which represents a rigid perfectly plastic body, is replaced with a modified Kepes slider that accounts for two-phase plasticity. Additionally, to ensure the threshold force for the reverse transformation is lower than that for the martensitic transformation, a parallel spring with negative stiffness is introduced (Figure 7a).

The modified structure incorporating internal hysteresis loops (Figure 7a) is introduced, featuring a two-phase plasticity slider and a negative stiffness spring. The hysteresis loops in Figure 7b visually demonstrate the formation of internal hysteresis loops due to incomplete phase transformations, highlighting the realistic behavior captured by this advanced model variant.

The stiffness’ parameters of this rheological structure is described by following relations:

(12) $$\begin{array}{l} \alpha_{1}=\mathrm{arctg}\left(k_{2}\right)\\ \alpha_{2}=\mathrm{arctg}\left(\frac{k_{2}\left(1-\beta \right)T_{0}^{kp}\left(x_{tr}\right)}{k_{2}+\left(1-\beta \right)T_{0}^{kp}\left(x_{tr}\right)}\right)\\ \beta \in \langle 0,1\rangle \end{array}.$$

The constitutive relationships for this material model have been derived and discussed in [22].

Each presented rheological model variant offers unique advantages and potential trade-offs regarding accuracy and computational complexity. The basic SMA model (Section 2.1) provides simplicity and computational efficiency, making it suitable for routine analyses involving a large number of elements. Introducing kinematic hardening (Section 2.2) improves the realism of the material response at the cost of a moderately increased computational effort due to the additional internal variable and equations. The locking mechanism (Section 2.3) and variable stiffness models (Section 2.4) further enhance the model accuracy in capturing specific material behaviors but require increased computational resources, due to more complex conditional logic and additional numerical parameters. The internal hysteresis loop model (Section 2.5) provides the most accurate representation of complex SMA behaviors, but with significantly higher computational complexity, requiring additional internal variables and more elaborate numerical schemes. Thus, the choice of model should be aligned with the specific application requirements and computational resources available.

## 3. Numerical Simulation

### 3.1. Description of Analyzed Example

**Figure 8:**
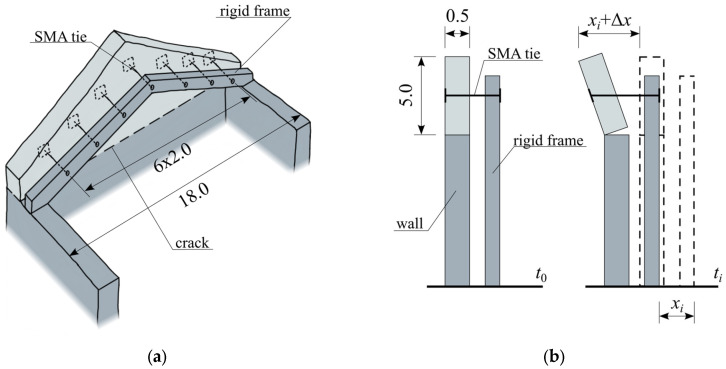
Analyzed strengthening concept: (**a**) general sketch; (**b**) assumed theoretical propagation of ground displacement to the modeled structure. Dimensions of the analyzed structure given in meters.

**Figure 9:**
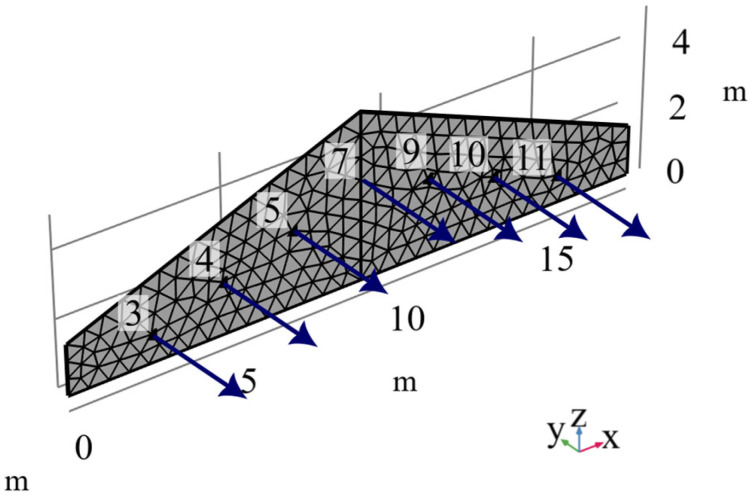
Model of the analyzed wall fragment with load application points marked and finite element mesh.

The formerly presented SMA modeling strategy was implemented in a simulation of the strengthening of a model historical masonry structure—a tympanum, which is a typical crowning element of historic building façades. Based on existing applications [11,12,13], this strengthening method involves the construction of a rigid frame behind the tympanum, which is then connected to the historical structure by a series of short tie rods incorporating SMA components (Figure 8a). In the modeled example, a masonry tympanum was assumed with a total width of 18.0 m, a height (above the building walls) of 5.0 m, and a thickness of 0.5 m. The analysis assumed that under seismic excitation, failure would occur through the formation of a local failure mechanism, characteristic of such structural elements, involving the out-of-plane overturning of the tympanum at the level of the gable wall tops. During the development of this mechanism, horizontal cracking of the wall occurs, enabling the tympanum to rotate independently from the remaining portion of the wall. In the adopted structural model, only the triangular part of the masonry was considered, assuming the rigid-body motion of the lower part of the wall together with the ground. Due to the cracking induced by the formation of the failure mechanism, a rotational degree of freedom was assumed at the base of the modeled façade element. The schematic representation of the soil–structure displacement transfer is shown in Figure 8b. For the finite element model developed in COMSOL Multiphysics 6.1 (Figure 9), shell elements were used. Given that the simulation focused on the analysis of the reinforcing tie rods, a simplified linear-elastic model of the masonry structure was adopted. The mechanical properties were defined based on the values reported in the literature [23] and are summarized in Table 1.

As an example of a high intensity earthquake, the Loma Prieta earthquake was adopted as the excitation in the simulation. This earthquake struck the state of California (USA) on 17 October 1989. The time history of the ground displacements recorded during the event is presented in Figure 11 (dashed line) [24]. The load was applied at the bottom edge of the modeled structural element, in accordance with the assumption of the rigid-body motion of the lower part of the building.

### 3.2. SMA Model Assumed in the Simulation

**Figure 10:**
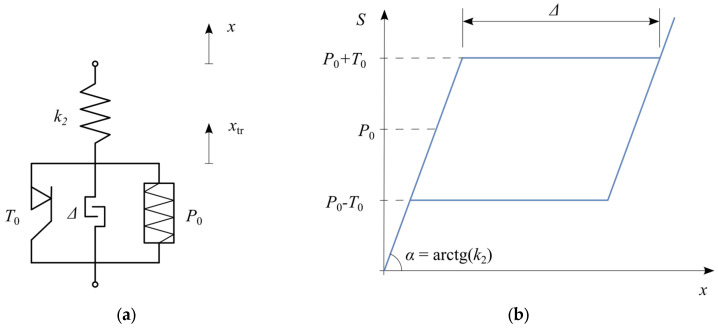
SMA model used for the strengthening simulation: (**a**) rheological structure; (**b**) hysteresis loop.

In the modeled case, seven ties were assumed to connect the façade to the rigid frame, spaced at intervals of 2.0 m (Figure 8a). Each tie incorporated devices containing eleven SMA wires with a diameter of 1.5 mm, corresponding to a SMA total cross-sectional area of 0.19 cm^2^. The geometrical parameters of the SMA elements of the ties were selected to satisfy the requirement of the maximal acceptable horizontal displacement, set as 25% of the instability displacement [25]. The loads transmitted by the ties to the structure were modeled as concentrated forces applied at the anchorage points (Figure 9). To derive the equations describing the relationship between the displacement of the anchorage point and the force transmitted by the tie to the structure, a modified SMA model was used, as shown in Figure 10. The introduction of a locking element into the base model described in Section 2.1 requires a modification of Equation (3) to the following form of Equation (13).

(13) $$\begin{array}{l} {\dot{x}}_{tr}=f_{SMA}\left(\dot{x},x,x_{tr}\right)\\ f_{SMA}=\left\{\begin{array}{l} \dot{x}\;\mathrm{if}\;\left|S\right|=P_{0}+T_{0}\;\mathrm{and}\;S\dot{x}>0\;\mathrm{and}\;\left|x_{\mathrm{tr}}\right|<\Delta \\ \dot{x}\;\mathrm{if}\;\left|S\right|=P_{0}-T_{0}\;\mathrm{and}\;S\dot{x}<0\;\mathrm{and}\;Sx_{\mathrm{tr}}>0\\ 0\;\mathrm{otherwise} \end{array}\right. \end{array}$$

The values of the SMA material parameters, as well as the corresponding parameters characterizing the elements of the rheological model, are presented in Table 2. The previously adopted dimensions of the applied SMA elements were taken into account in order to determine the values of the rheological model parameters. It is important emphasize that each rheological element’s parameter is directly related to the material parameters that are possible to measure during the simple mechanical test of the SMA sample:

- $k_{2}$ is related to the material stiffness (Young’s modulus E),
- $T_{0}$ and $P_{0}$ are the force levels related to the stress levels on which phase transformation occurs ($\sigma_{AM}$ and $\sigma_{MA}$),
- Δ is related to the strain (deformation) after the phase transformation is finished ($\varepsilon_{M}$).

### 3.3. Simulation Results

Using the capabilities of COMSOL Multiphysics, which allows the user to define physical phenomena through direct programming of ordinary differential equation systems (ODE Interface), the force values were calculated at each time step based on the displacements computed (within Shell interface) by the Structural Mechanics module. The generalized-α solver was used for 40 s of total analyzed time with a 0.001 s manual time step.

**Figure 11:**
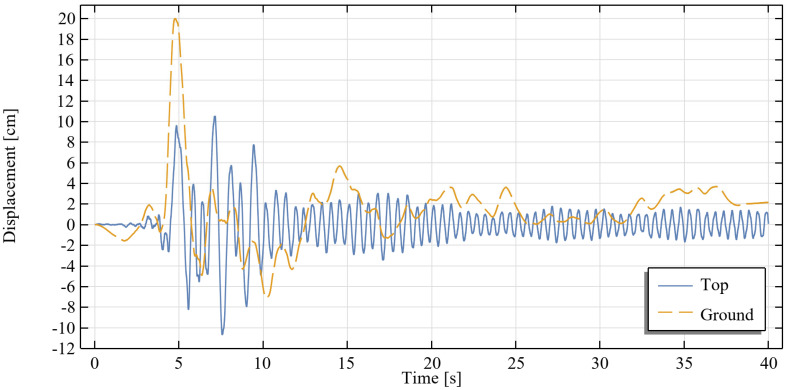
Time history of the displacements of the apex of the modeled structure relative to the ground and the ground displacements.

**Figure 12:**
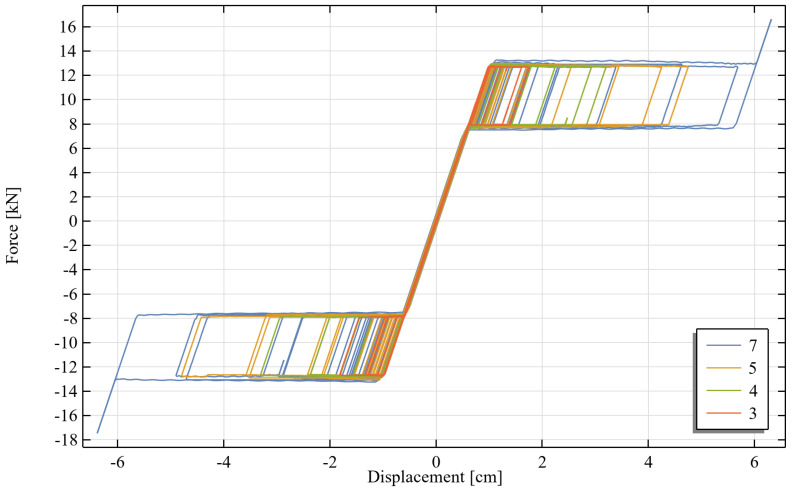
Hysteresis loops of SMA ties at different levels (level designations according to Figure 9).

The analysis results are presented in the form of the time history of the displacement of the top of the structure relative to the ground (Figure 11) and the hysteresis loops for each tie level (Figure 12).

Analysis of the development of the hysteresis loops as a function of the intensity of excitation clearly reveals different operational stages of the SMA ties. In the case of small displacements, the tie operates within the elastic range, without initiating the phase transformation of the SMA. For larger displacements, the phase transformation into martensite begins, resulting in an increase in strain without a corresponding increase in force (visible as a nearly horizontal plateau in Figure 12). Deformation is subsequently recovered during the reverse phase transformation. Under the highest intensity of excitation, the SMA undergoes full transformation into martensite, leading to a re-stiffening of the material.

## 4. Discussion

The presented formulation of the SMA constitutive equations in the form of a force–displacement relationship using rheological structures enables the derivation of a system of explicit ordinary differential equations. Such a system of equations can be implemented in any computational software, as demonstrated using COMSOL Multiphysics 6.1.

The numerical analysis results confirm the correctness of the phenomenological formulation of the SMA model. The presented model accurately describes the macroscopic phenomena occurring during the phase transformations induced by the deformation of SMA structural elements (the superelastic effect). It should be emphasized that all parameters defining the model have a direct correspondence to the physical quantities that are measurable in mechanical tests of SMA materials.

In order to compare the strengthening technique incorporating SMA elements, an alternative conventional strengthening method was simulated for the same structural model under identical loading conditions. In this second scenario, the ties were assumed to be made of steel. The diameters of the rebars were initially determined based on the same limitation of the maximum allowable horizontal displacements of the structure. This criterion could be satisfied using small-diameter steel rebars, but it would result in very high tensile stresses within the ties. An additional requirement was imposed to ensure that the stress level in the steel would not exceed its yield strength, which corresponds to the reversible elongation of ties, such as in the case of SMA ties. As a result, the diameter had to be increased to 18 mm, which led to a significant increase in the overall stiffness of the structure and a further reduction in the displacements. The results of the comparative analysis are presented in Table 3.

While the higher stiffness of the steel-strengthened structure effectively reduced the horizontal displacements, it also caused significantly greater point loads to be transferred to the structure. Masonry, especially historical structures characterized by low cohesion, are particularly vulnerable to this type of load. The incorporation of SMA allows the precise determination and control of the level of force that is transferred to the structure by the ties. The level of force that triggers the phase transformation can be designed for a specific structure and expected load, but it does not jeopardize the safety of structure if, in any case, the excitation exceeds the design load, which could happen in case of steel ties. It is a result of the reversible character of the phase transformation, which is responsible for the characteristic plateau, opposite to the yielding of steel. Moreover, during the phase transformation, the energy dissipation occurs, which enhances the vibration damping of a structure.

In the presented form, the SMA material models simulate the superelastic behavior in a specific temperature (isothermal condition). The further development of these models will be oriented toward the implementation of the rheological elements’ parameters’ dependency on temperature. Another limitation of this modeling approach is its application to a one-dimensional case, which is suitable for ties’ analysis but not for more complex applications. In such a case, a proper yielding criterion has to be implemented. For instance, Auricchio [26] introduced a Drucker–Prager equivalent stress typical for soil models. The presented modeling approach is limited to moderately large deformations. For large deformations adequate and energetically consistent measures of strain and stress have to be considered.

## 5. Conclusions

The presented equation-based SMA modeling approach, formulated using rheological structures, effectively captures superelastic behaviors critical for structural reinforcement applications. The numerical analysis of a historical masonry structure subjected to seismic excitation (Loma Prieta earthquake record) demonstrates the clear advantages SMA-based ties allow to satisfy the safety requirement of the maximal horizontal displacement, with significant force limitations compared to conventional steel ties. Within the assumption of the recoverable ties’ deformation, the force transferred to the structure by an SMA tie is approximately 86% lower than a steel tie. The possibility of control over the force transferred to a historical structure is an important benefit in case of their retrofitting. This could be obtained by the proper selection of geometrical parameters of the SMA ties (such as an area of a tie’s cross section), which leads to the controllable deformation of a historical structure without endangering its structural safety (re-stiffening of ties under higher-than-expected excitation).

While the numerical outcomes affirm the theoretical predictions and validate the modeling strategy, further experimental investigations are necessary. Future work should include comprehensive laboratory tests under dynamic excitation to verify the model parameters and hysteresis characteristics experimentally. Moreover, additional research could explore the optimization of SMA tie arrangements and detailed parametric studies investigating varying seismic loading scenarios.

In summary, the developed approach offers clear engineering benefits, specifically a quantifiable improvement in structural performance, the enhanced predictability of SMA behaviors, and increased applicability due to simplified parameter identification. Further experimental validation and optimization studies will be crucial in advancing this promising seismic retrofitting technique in practical civil engineering applications.

## Figures and Tables

**Table 1:** Material parameters of masonry assumed in the simulation [23].

Density	Young Modulus	Poisson’s Ratio
ρ [kg/m^3^]	E [MPa]	ν [−]
1600	1200	0.2

**Table 2:** Material and rheological model parameters assumed in the simulation.

Material Parameters			
E [GPa] 66.8	$\sigma_{MA}$ [MPa] 650	$\sigma_{AM}$ [MPa] 410	$\varepsilon_{M}$ [%] 6
**Rheological model parameters**			
$k_{2}$ [kN/mm]	$T_{0}$ [kN]	$P_{0}$ [kN]	Δ [mm]
1.298	2.33	10.3	50.3

**Table 3:** Comparative analysis of strengthening techniques.

	SMA Ties	Steel Ties
Maximal horizontal displacement of the apex of the structure	10.68 cm	1.06 cm
Maximal force transferred to the strengthened structure by the topmost tie	17.47 kN	125.56 kN

## Data Availability

The original contributions presented in this study are included in the article. Further inquiries can be directed to the corresponding author.
